# Infertility in Mazandaran province - north of Iran: an etiological study

**Published:** 2011

**Authors:** Abbasali Karimpour Malekshah, Amir Esmailnejad Moghaddam, Narges Moslemizadeh, Sepideh Peivandi, Ayyub Barzegarnejad, Nadali Musanejad, Gholamali Jursarayee

**Affiliations:** 1 Department of Embryology, Cellular and Molecular Center, Imam University Hospital, Infertility Center, Mazandaran University of Medical Sciences, Sari, Iran.; 2 Department of Embryology, Infertility Center, Imam University Hospital, Mazandaran University of Medical Sciences, Sari, Iran.; 3 Department of Obstetrics and Gynecology, Infertility Center, Imam University Hospital, Mazandaran University of Medical Sciences, Sari, Iran.; 4 Department of Urology, Infertility Center, Imam University Hospital, Mazandaran University of Medical Sciences, Sari, Iran.; 5 Department of Embryology, Fatemeh Zahra Infertility Center, Babol University of Medical Sciences, Babol, Iran.

**Keywords:** *Primary infertility*, *Secondary infertility*, *Male factor*, *Ovarian dysfunction*, *Varicocel*

## Abstract

**Background: **The prevalence and etiology of infertility are not similar in different parts of the world. There are only few reports of this topic in Iran.

**Objective: **This study was conducted to determine the clinical patterns and major causes of infertility in Mazandaran province in north of Iran.

**Materials and Methods: **The medical records of 3734 consecutive couples attending two infertility clinics in Mazandaran province, from 2003 to 2008, were reviewed. The couples had not had a viable birth after at least 1 year of unprotected intercourse and were fully investigated.

**Results:** Of the entire samples, 78.7% had primary infertility and 21.3% had secondary infertility. The mean duration of infertility in couples was 5.7±4 years. The etiology of infertility in couples revealed; male factor in 38.9%, female factor in 34.7%, combined factors in 14.6% and undetermined cause in 11.8%.

**Conclusion:** In this study, delayed attendance of infertile couples to the infertility clinic was found. Therefore, there is a need to revise public health program on infertility to focus on the education and prevention of infertility and its risk factors.

## Introduction

 Infertility, defined as the inability of couple to achieve conception after at least 1 year of regular unprotected intercourse, is a common problem in the world and affects about 8-12% of couples (1). The levels and patterns of infertility apparently vary widely and also are different in developed countries compare to those in developing regions of the world (2).We found in our previous study, a prevalence of 13.2% of infertility in Mazandaran province - north of Iran (3). But Vahidi et al (2006) reported a prevalence of 24.9% of infertility around the country (4). Cultural, socioeconomic, health care practices and policies and environmental factors play a major role in the prevalence and etiology of infertility (5). Also the proportions of causes of infertility have changed over time (6). The most cost-effective approach to solving the infertility problem is prevention and education. Further research in both developed and developing countries is needed to understand the high prevalence and causes of infertility (7). There are only a few reports on this topic in Iran (8,9) Therefore, determination of the clinical patterns and a review of the major causes of infertility in north of Iran were the main objectives of this study. 

## Materials and methods

 In this descriptive study the medical records of 3734 consecutive couples attending two infertility centers in Mazandaran province, from 2003 to 2008, were reviewed. A complete history was taken and a complete physical examination was performed for all patients. In the men, particular attention was paid to the pattern of pubertal development, history of cryptorchidism, inguinal surgery, mumps, orchitis, testicular torsion, viral illness, and sexually transmitted diseases. The external genitalia were examined for number, site, and size of the testes and presence of varicocele and any other congenital or acquired defect. In the women, the emphasis in history was on the pattern of pubertal development, menstrual history, galactorrhea, any virilization or defeminition, and drug or hormone intake. Sexual history focused on libido, erectile function, frequency and timing of intercourse.

 Specific investigations performed for the male partner included semen analysis and hormone estimations. Semen analyses were performed according to the method described in the World Health Organization laboratory manual (10). 

 A detailed gynecologic examination was carried out to ascertain cervical cause of infertility such as cervical stenosis, abnormal cervical mucus, or diseases such as endometriosis. The specific investigations performed for the female partner included tests for documentation of ovulation (gonatorophins and steroids assays and ultrasonography), test for tubal patency, and laparoscopy (when indicated). Tubal patency was assessed hysterosalpinography. Laparoscopy was performed in some cases to study tubal diseases and to look for endometriosis.

**Statistical analysis**

 Descriptive statistics and statistical analysis was performed using the software package SPSS 13. 

## Results

 The total of 3734 infertile couples were assessed in this study. Of these, 2941 (78.7%) couples had primary infertility and 793 (21.3%) had secondary infertility. The mean age (±SD) of the men was 33±7 (range, 21-64 years), and the mean age (±SD) of the women was 29 ± 6 (range, 17-47 years). The mean duration (±SD) of infertility at the time of presentation to infertility clinic was 5.7±4 years.

 The distribution of causes of infertility in study couples is shown in Tables I and II. Of the entire of infertile couples, 38.9%, 34.7% and 14.6% had infertility due to at least a male factor, a female factor and both couple involvement respectively. In 11.8% of infertile couples, the cause remained unexplained (Table II).

 Semen factor was the most common identifiable etiologic factor, documented in 1884 (50.5%) couples. Among these men 804, (42.7%) had varicoceles. Other important causes were vas deferens agenesis (1.2%), cryptorchidism (0.5%), and testicular torsion (0.1%). In 1045 (55.5) men, no cause could ascertained. Ovarian dysfunction was the second most common etiologic factor of infertility, recognized in 1074 (28.8%) women. Of these 610 (56.8%) women had polycystic ovary syndrome. Tubal factor was the other main etiologic factor of infertility, recognized in 553 (14.8%) women (Table I).

**Table I:** Distribution of the causes of infertility among the male and female partners of infertile couples in Mazandaran

**No. of couples (%)**	**Etiology of infertility**
1884 (50.5)	Semen factor
114 (3.1)	Miscellaneous male factors (hypospadias, retrograde ejaculation, coital problem)
1074 (28.8)	Ovarian dysfunction
553 (14.8)	Tubal factor
85 (2.3)	Endometriosis
71 (1.9)	Uterine factor
84 (2.2)	Pelvic adhesion
3734*	Total

* Note: This is a real number of infertile couples which includes unexplained infertility and not cumulative of above categories.

**Table II:** Causes of infertility by gender in infertile couples of Mazandaran

**Number of couples (%)**	**Categories**
1453 (38.9)	Male factor only
1295 (34.7)	Female factor only
545 (14.6)	Both partner involved
441 (11.8)	No causes found in both (unexplained)
3734 (100)	Total

## Discussion

 In this study a relatively high frequency of primary infertility (78.7%) was observed. While secondary infertility was determined in 21.3% of couples. This finding confirms other regional and national studies in this aspect (11,12). The most comprehensive study of infertility- a WHO study of 5800 infertile couples seeking help at 33 medical centers in 22 developed and developing countries- found that most infertile couples around the world suffer from primary infertility (13). Sub-Saharan Africa is an exception, in this region most couples (52%) suffered from secondary infertility (13). A study was conducted by Orhue and Aziken (2007) in Nigeria confirmed WHO report and showed even greater discrepancy in parity distribution. According to their findings the percentage of secondary infertility is 85.7% among infertile couple of Nigeria (14). Latin America also had a relatively high rate of secondary infertility (40%). In contrast, only 23% of infertile couples in Asia and 16% in North Africa suffered from secondary infertility (13). Mongolia is an exception in Asia with 43.7% of secondary infertility (15). The results of WHO study suggest that repeated pregnancy play a greater role in the etiology of infertility in Africa and Latin America, while repeated abortions are important in Asia and developing countries (16). In many cases in the developing countries, infertility in women results from untreated pelvic inflammatory disease (PID), a sequel of an sexually transmitted diseases (STDs) or other reproductive tract infections (2,15). 

 The mean age of the male and female partners were 33±7 years and 29±6 years respectively which are almost similar to some other reports from national studies (11,12) and those from developing countries (15,17-19). 

 The causes of infertility can be divided into four major categories: the female factor, the male factor, combined factors and unexplained infertility. In our study, male factor consists 38.9% of infertility, female factor occurred in 34.7% of the infertile couples, in 14.6% of the couples both partners involved and in 11.8% of couples no cause could be ascertained. In a field study in central part of Iran (Yazd province), Aflatoonian et al (2009) reported female factor as the main cause of infertility (57.7%) (8). On the contrary, in another study on infertile couples referred to Royan Institute in Tehran, male factor was found to be the most important etiologic factor (50.5%) (9). Different sampling and laboratory analytical methods may be attributable to these controversies. Most studies have reported a male factor in 20-40%, female factor in 30-55%, both couple involvement in 5-35% and undetermined cause in 5-15% (8, 11**, **12, 19-24). However, the results of some studies are quite different, for example a study conducted in Western Siberia, found that male factors were responsible for infertility in 6.4%, and female factors were responsible in 52.7% of couples (25). While differences in data sources and analyses make it difficult to accurately measure or compare infertility rates, it is clear that the level and causes of infertility vary widely, both among and within countries (2). Approximately only 5% of the infertility incidence in couples is due to endocrinological, anatomical, genetic an immunological problems leaving about 95% of the infertility preventable. These preventable conditions, including: STD, parasitic diseases, health services and exposure to environmental toxic substances. (2).The factors that contribute to these conditions vary from region to region (2, 5, 9, 26, 27).

 The duration of infertility found in our study was longer (5.7±4 years) than that in developed countries (19), as well as some developing countries (19). It is however, similar to that in some other developing countries e.g. Indian Kashmir (17) and Iran (11, 12). Infertile couples do not usually present in time to the infertility clinics due to inadequate general knowledge regarding infertility and about the presence of special centers in the country. There are state owned as well as private general and special hospitals, but almost all of the medical doctors have private single special small clinic without any paraclinical facilities at the same place. These small private clinics are available to the patients without any waiting list and only by paying a visit charge of less than $15. They may (at least in some cases) offer advice or treatments without conducting a complete evaluation, sometimes even without seeing both partners. This situation makes the patients curious to try many such clinics especially when they do not obtain suitable results from the first prescription. This, in turn, makes a considerable confusion for the patients and a substantial delay before attending to an infertility clinic.

 Based on these data it is recommended to take measures to improve the referral system, fertility health education and implementing infertility prevention programs. Moreover, merging all those small private clinics into private, public or states owned hospitals is suggested, in order to assure timely, proper and adequate treatment. 

## References

[1] World Health Organization (1991). Infertility: A tabulation of available data on prevalence of primary and secondary infertility.

[2] Kds A, Nguyen T (1997). Infertility in developing countries. Reproductive Health Outlook.

[3] Moghaddam A, Karimpour Malekshah A, Talebpour Amiri F, Taringou F (2000). The prevalence of infertility in center region of Mazandaran province in 1999. Journal of Mazandaran University of Medical Sciences.

[4] Vahidi S, Ardalan A, Kazem m (2006). The epidemiology of primary infertility in the Islamic republic of iran in 2004-2005. Journal of Reproduction and Infertility.

[5] Leke RJ, Bassol-Mayagoitia S, Bacha AM, Grigor KM (1993). Regional and geographical variation in infertility: effects of environmental, cultural and socioeconomic factors. Environ Health Perspect.

[6] Terava AN, Gissler M, Hemminiki E, Luoto R (2008). Infertility and the use of infertility treatments in Finland: prevalence and socio-demographic determinants 1992-2004. Eur J Obstet Gynecol Reprod Biol.

[7] Wyshak G (2001). Infertility in Americian college alumnae. International Journal of Gynecol Obstet.

[8] Aflatoonian A, Seyedhassani SM, Tabibnejad N (2009). The epidemiological and etiological aspect of infertility inYazd province of Iran. Iranian Journal of Reproductive Medicine.

[9] Safarinejad M (2008). Infertility among couples in a population-based study in Iran: prevalence and associated risk factors. Int J Androl.

[10] World Health Organization (1999). WHO laboratory manual for the examination of human semen and sperm-cervical mucus interaction.

[11] Esmeilzadeh S, Farsi M, Nazari T (2002). The cause of infertility in the patients referring to Babol township fatemeh Zahra infertility center from May 1996 to May 1998. Journal of Mazandaran University of Medical Sciences.

[12] Kamali M, Kashfi F, Baghestani AR, Kashani H, Tavajohi SH, Amirchaghmaghi E (2006). The epidemiologic survey on causes of infertility in patients refered to Royan institute. Medical Journal of Tabriz University of Medical Sciences.

[13] Cates W, Farley TMM, Rowe PJ (1985). Worldwide patterns of infertility. Is Africa different?. Lancet.

[14] Orhue A, Aziken M (2008). Experience with a comprehensive university hospital-based infertility program in Nigeria. Int J Gynaecol Obstet.

[15] Bayasgalan G, Naranbat D, Tsedmaa B, Tsogmaa B, Sukhee D, Amarjargal O, Lhagvasuren T, Radnaabazar J, Rowe PJ (2004). Clinical patterns and major causes of infertility in Mongolia. J Obstet Gynaecol Res.

[16] World health organization (1987). Infections, pregnancies and infertility: Perspectives on prevention. Fertil Steril.

[17] Zargar AH, Wani AI, Masoodi SR, Laway BA, Salahuddin M (1997). Epidemiologic and aspects of primary infertility in the Kashmir region of India. Fertil Steril.

[18] Omu AE, Ismail AA, AL-Qattan F (1999). Infertility in Kuwait. Int J Gynaecol Obstet.

[19] Cates W, Farley TM, Rowe PJ, Rowe PJ, Vikhlaeva EM (1988). Patterns of infertility in the developed and developing world. WHO symposium on diagnosis and treatment of infertility.

[20] Isaksson R, Tiitinen A (2004). Present concept of unexplained infertility. Gynaecol Endocrionol.

[21] Forti G, Krausz C (1998). Evaluation and treatment of the infertile couple. Clin Endocrinol Metabol.

[22] Thonneau P, Marchand S, Tallec A, Ferial ML, Ducot B, Lansac J (1991). Incidence and main causes of infertility in a resident population (1,850,000) of three Franch regions (1988-1989). Hum Reprod.

[23] Hull MG, Glazener CM, Kelly NJ, Conway DI, Foster PA, Hinton RA, Coulson C, Lambert PA, Watt EM, Desai KM (1985). Population study of causes, treatment and outcome of infertility. Br Med J (Clin Res Ed).

[24] Rahim R, Majid SS (2004). Aetiological factors of infertility. JPMI.

[25] Philippov OS, Radionchenko AA, Bolotova VP, Voronovskaya NI, Potemkina TV (1998). Estimation of the prevalence and causes of infertility in western Siberia. Bull World Health Organ.

[26] Inhorn Mc, Buss KA (1994). Ethnography epidermiology and infertility in Egypt. Social Science and Medicine.

[27] Ericksen K, Brunette T (1996). Patterns and predictors of infertility among African women: A cross-national survey of twenty seven nations. Social Science and Medicine.

